# Using Picture and Text Schedules to Inform Children: Effects on Distress and Pain during Needle-Related Procedures in Nitrous Oxide Sedation

**DOI:** 10.1155/2015/478503

**Published:** 2015-12-22

**Authors:** Merja Vantaa Benjaminsson, Gunilla Thunberg, Stefan Nilsson

**Affiliations:** ^1^Södra Älvsborg Hospital, 504 55 Borås, Sweden; ^2^DART-Center for AAC and AT, The Queen Silvia Children's Hospital, Sahlgrenska University Hospital, 411 04 Gothenburg, Sweden; ^3^Faculty of Caring Science, Work Life and Social Welfare, University of Borås, 501 90 Borås, Sweden; ^4^Institute of Health and Care Sciences, University of Gothenburg, 405 30 Gothenburg, Sweden

## Abstract

During hospital visits, children often undergo examinations and treatments that may involve an experience of pain and distress that is also connected to the staff's treatment. The United Nation's Convention on the Rights of Persons with Disability advocates the use of Universal Design. One way of implementing this idea within paediatric nursing is to increase the use of pictorial supports, and the few studies that have been published show promising results. The aim of this study was to do a comparison between two groups of children in regard to the pre- and postconditions of implementing an intervention including staff instruction and the use of pictorial support. The support consisted of a visual schedule with pictures and text, used both preparatory to and during the hospital visit. One hundred children aged 5–15 (50 children during the preinterventional data collection and 50 children postinterventionally) reported pain intensity and distress during needle-related procedures in nitrous oxide sedation. The results showed that the intervention had a positive effect in significantly lowering the level of preprocedural distress. The results showed that the pain intensity was also lowered however not reaching statistical significance. This confirms other positive research results on the use of visual supports within paediatric care, a topic that has to be further studied.

## 1. Introduction

The United Nation's (UN) Convention on the Rights of the Child states that all children should be provided with information that is adequate according to their cognitive, physical, and social development, including the right to get information that is important for health and well-being [1]. Article 12 of the UN's Convention on the Rights of the Child stresses that health care professionals “*shall assure the child who is capable of forming his or her own views the right to express those views freely in all matters affecting the child*” [1]. Because of this, it is important to find ways of giving information and providing strategies that are suitable for a child and his/her family. However, a systematic review found that few paediatric clinical practice guidelines for acute procedural pain were clear, concise, and specific or given in an easy-to-follow format; that is, most guidelines for children were in need of improvement [2]. Earlier research has also identified a lack of strategies in nursing literature about how to facilitate communication between the nurse and the child [3].

Communicative rights are reinforced in the UN's Convention on the Rights of Persons with Disability (CRPD), a document that stresses a child's right to accessibility and that health care staff must take all measures to ensure that persons with disabilities have access to information on equal conditions as others have [4]. The use of Universal Design is also put forth (Articles 2, 3, and 21 [4]). When it comes to information and communication, this means that augmentative and alternative communication (AAC) forms should be used to make health care accessible to as many individuals as possible [4]. Hitherto, parents of children with communicative disabilities have experienced a lack of access to information when their children visit the hospital [5, 6]. This stresses the need for adequate access to communication tools, as well as access to individual health information [5]. To achieve this goal, it is necessary to give nurses education about how to use these communication tools [5, 6]. There is a need for educating nurses in using simple, functional, and generic augmentative communication strategies [5]. However, there is a lack of strategies in the literature and a need for research into effects [5]. This lack of knowledge might cause unnecessary suffering for a child [3]. The few studies that have been published point to good results [7, 8].

During hospital visits, children often undergo examinations and treatments that may involve an experience of pain and distress. Procedural pain, which often means a pain induced by a stick, is mediated primarily through nociceptive A-delta fibres [9]. Pain from A-delta fibres is treated effectively using local anaesthetics, such as a Eutectic Mixture of Local Anaesthetics (EMLA) (lidocaine 25 mg/g and prilocaine 25 mg/g) [10]. When a procedure is expected to give children moderate pain and distress, local anaesthetics may be insufficient as the only drug in pain management. This is especially true if a child also worries about how he or she will manage the situation. In these cases, it is effective to add a drug that is both analgesic and sedative. Accordingly, 50% nitrous oxide and 50% oxygen in combination with EMLA are often effective to reduce the pain caused by injections [11]. Acetaminophen and cyclooxygenase (cox) inhibitors (ibuprofen, diclofenac, and ketorolac) may relieve the pain that can be traced to the inflammation that may occur afterwards, but the evidence for this is low [12].

As stated above, a child's right to be provided with adequate information and to express himself/herself is important also in needle-related procedures. When nurses lack the ability to explain what is going to happen, this decreases the possibility of calming a child. At times, this situation may make it necessary to overpower and restrain a child during a procedure [13]. Sometimes, a needle-related procedure even has to be cancelled, which generates extra costs for the organization and decreases the child's will to come back voluntarily to the hospital.

The project KomHIT—communication in care settings using communicative support and IT—has the overall purpose of improving the communicative rights of children during paediatric or dental care situations according to the CRPD, focusing on the use of AAC as Universal Design (Articles 2, 3, and 21 [4]). The hypothesis is that when care staff have better knowledge about communication and routinely use AAC in information and communication processes to meet the rights of children with a communicative disability, the communication situation and quality of care are increased for all children. Furthermore, not only are the numbers of individuals who have communicative problems during care situations limited to children with a disability and their parents but they also include a large proportion of the population [14]. For example, young children, being at early linguistic levels, and their parents have problems, as have immigrant families that are not acquainted with the often complicated language used by medical staff and/or medical culture [14]. KomHIT, therefore, tries to implement AAC strategies, mainly in the form of pictorial supports, to be used generally and routinely to all children within paediatric care. The first studies within this project have shown promising results [15].

## 2. Aim

The aim of this study was to evaluate the KomHIT-intervention using education to the staff and pictorial supports as Universal Design for communication and information implemented at a paediatric unit. More specifically, the purpose was to investigate whether instruction to the staff and the use of a visual schedule, provided before and during the hospital visit, decreased distress and pain intensity in children who underwent needle-related procedures in nitrous oxide sedation.

## 3. Methods

### 3.1. Study Design

A nonrandomised clinical trial was conducted [16] in which data were consecutively collected from groups of participants.

### 3.2. Participants

One hundred children participated: 50 children during the preinterventional data collection (26 boys and 24 girls; mean 9.64 years) and 50 children after the intervention was implemented (26 boys and 24 girls; mean 9.12 years) (Table 1).

Universal Design was used also in regard to the inclusion of participants. This means that all children were invited; that is, no child was excluded due to disability. According to the medical records, five children in the preinterventional group and eight children in the postinterventional group had a communicative disability. In the preinterventional group, three boys and one girl had Attention Deficit Hyperactivity Disorder (ADHD), and one girl had intellectual disability. In the postinterventional group, five boys had ADHD, one girl had intellectual disability, and two boys had specific language impairment. No statistical calculation was conducted due to the low number of participants with a communicative disability.

### 3.3. Instruments

#### 3.3.1. Distress

The Facial Affective Scale (FAS) is a self-reported scale with nine faces that assesses a child's distress, ranging from 0.04 (happiest feeling possible) to 0.97 (saddest feeling possible) [17]. Distress was reported on three occasions, that is, before, during, and after each needle-related procedure.

#### 3.3.2. Pain Intensity

The Coloured Analogue Scale (CAS) is a self-reported scale that assesses a child's pain intensity, ranging from zero (no pain) to ten (worst possible pain). This scale is designed to provide gradations in colour, area, and length, reflecting different values of pain intensity [17]. Pain intensity was reported on three occasions, that is, before, during, and after each needle-related procedure.

### 3.4. Intervention

All children underwent a needle-related procedure in nitrous oxide (50% nitrous oxide/50% oxygen) sedation (Table 1). Needle-related procedures are often defined as injections, venepunctures, sutures, and lumbar punctures [18]. In this study, the concept of needle-related procedures also included pin-drawing, which is associated with similar pain and distress as other needle-related procedures [19]. No changes were conducted in the guidelines that were used for the pain management. Based on the guidelines, the children got acetaminophen, cox inhibitors (ibuprofen, diclofenac), and EMLA.

**Figure 1:**
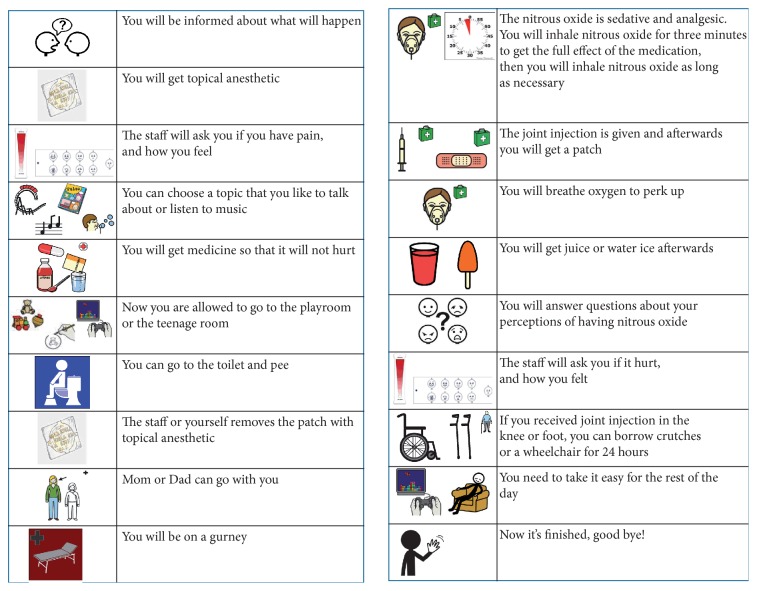
A visual schedule.

The intervention included a (a) preparatory phase and a (b) procedural phase. During the preparatory phase (a), a visual schedule was sent home by post to the family, depicting and in short text explaining the different steps of a nitrous oxide process (Figure 1). If the procedure was performed acutely, the family received the same visual schedule about two hours before the procedure. In both cases, the parents were instructed to go through the material with the child. The procedural phase (b) included the use of the same visual schedule during the nitrous oxide process at the hospital ward. The staff supplied each child with a schedule and pointed to the pictures when verbally informing the family of everything that was going to happen. The family used the information throughout the procedure. This meant that the child had control over how many steps were finished and how many steps were left to undergo. If something had to be changed, the visual schedule also could be used as a resource for understanding.

### 3.5. Data Collection

Data were collected preinterventionally between May and November 2013. Children were invited consecutively until 50 children had agreed to participate in this study. The intervention was introduced after the completion of this first data collection. After the preinterventional phase, the nurses were given education, totalling two hours, including both a theoretical background and practical training. The theory covered knowledge about communicative rights, the idea of Universal Design, communicative disabilities, and augmentative communication strategies. The practical training included the use of the pictorial supports within role-plays. When the intervention had been implemented, a second data collection with another 50 participants was conducted. The data were collected between November 2013 and July 2014.

### 3.6. Data Analysis

The statistics were calculated using IBM SPSS Statistics for Windows, version 22. Statistical significance was considered if *p* < 0.05. Primary outcome was the FAS, and a power calculation showed that each group needed to be at least 33 participants. The hypothesis was that the FAS should decrease from the mean score of 0.68 to 0.54, and the standard deviation was assumed to be 0.20. This assumption was based on the use of the FAS in an earlier study [20]. The CAS and the FAS have been validated in earlier studies using parametric statistics [17], which led to the selection of parametric statistics in this study.

### 3.7. Ethical Considerations

The study was carried out according to the rules and recommendations of the regional ethics committee. This means that the benefits of the study were considered greater than the risks. The children underwent this procedure, whether or not they decided to participate in the study, meaning that the situation was not arranged due to this study. Written information was provided to the parents and was supplemented with verbal information to the children. The voluntary nature of the study, as well as the right to withdraw from the study at any time without any explanation or consequence, was highlighted. If the children agreed to participate, the parents were asked for written consent.

## 4. Results

### 4.1. Distress

It was seen that the children were distressed before the procedure (Table 2) but that the children that were provided with the visual schedule reported significantly lower levels on the Facial Affective Scale (FAS) (mean 0.41) compared to the case with the preinterventional group (mean 0.52). The FAS scores during and after the procedure were low in both groups (mean ≤ 0.33). The children with a communicative disability (in both groups) reported a mean value of 0.60/0.44 before the procedure, 0.48/0.23 during the procedure, and 0.24/0.16 after the procedure on the FAS.

### 4.2. Pain Intensity

The level of self-reported pain intensity (CAS) before, during, and after the procedures turned out to be low in all children (mean ≤ 1.74). There were no differences between the two groups reaching statistical significance before, during, or after the procedure. However, the trend was that the CAS scores were lower in the intervention group both before (mean 0.62 compared to 1.19) and after (mean 0.42 compared to 0.90) the procedure (Table 2). The children with a communicative disability (in both groups) reported a mean value of 2.40/0.69 before the procedure, 2.40/1.38 during the procedure, and 0.40/0.81 after the procedure on the CAS.

## 5. Discussion

The aim of this study was to evaluate the KomHIT communication intervention, encompassing education to the staff and the use of pictorial supports as Universal Design for communication and information. Whether this intervention could reduce the reported pain intensity and distress in children who underwent needle-related procedures in nitrous oxide sedation was studied.

The level of pain intensity as measured by the CAS showed to be low in both groups (mean ≤ 1.74), which means that the nitrous oxide sedation did help the children in their pain management, regardless of the communication intervention. However, this study had no control group without nitrous oxide sedation, so no conclusions could be drawn in this regard. Comparing the two groups of children in this study, it was seen that the children who had access to the KomHIT communication intervention reported a lower intensity of pain, however, one not reaching a level of statistical significance. A future study including larger groups of participants might prove if this trend is significant. It would also be interesting to compare nitrous oxide sedation with the use of pictorial support. In another study within the KomHIT project, it was seen that the number of children who were in need of premedication during day surgery was reduced when the KomHIT communication intervention with pictorial supports was introduced [21].

The results on the FAS scale where the children reported their feelings of distress before, during, and after the procedure showed that the children's preprocedural stress was significantly reduced when the KomHIT communication intervention was implemented. The levels of distress during and after the procedure were low for both groups, which again points to a positive effect of the nitrous oxide sedation. As already has been suggested, a future study should be done that compares the use of pictorial supports to nitrous oxide sedation. The possibility of identifying groups of children who can cope well with the use of pictorial supports, and therefore do not need nitrous oxide sedation, is highly relevant due to both costs and the negative pollution effects of nitrous oxide [22].

Taken together, the results in this study showed that the KomHIT communication intervention had a positive effect in reducing distress before the nitrous oxide sedation. Some other positive trends, such as reduced pain before and after the procedure, were also seen but could not be proved significant. These results confirm the earlier positive reports of the use of pictorial supports [7, 8]. The mechanisms behind these results are not yet fully known, and, in this study, it is impossible to separate the possible effects of staff education from the effects of having access to pictorial supports. But, most possibly, pictorial supports assist a child in creating a concrete idea of what will happen and so in bringing a child a sense of control in a situation that otherwise could be frightening for him or her. Achieving a sense of control is one of the most commonly used strategies for children in a needle-related procedure [23]. The child's feeling of control is dependent on the people around him or her, and this social support will be necessary for the child to gain a sense of control [24]. In general, the use of cognitive strategies to control pain will increase with age [25]. Children can view themselves as active agents in pain relief, and, to make this possible, they have to be informed about the procedure [26].

Nurses also have to fulfill the requirements of the conventions that come from the UN and that stress that all children should have access to information about their activities at a hospital [1, 4]. Hitherto, there has been little research in the context of paediatric care that provides nurses with evidence-based strategies when it comes to pictorial supports or other AAC strategies [27]. However, this study gives an example of an AAC strategy that has an effect on children's distress, at least in regard to procedures in nitrous oxide sedation.

### 5.1. Methodological Considerations

This study was not a randomised clinical trial, which was a weakness in regard to scientific control. However, the type of intervention that was evaluated also included staff education and communication, which means that it would have been tricky or impossible for nurses to change behaviour between different children. Additionally, earlier research has shown small differences among the results of randomised versus nonrandomised studies [28]. Another weakness in the study design was that children with and without a communicative disability were not separated in different study groups and that the children with a communicative disability were few in total (*n* = 13). This means that the results mainly tell us something about children without a communicative disability. Furthermore, it is not possible to separate the effects of the education about communication from the use of visual supports. These two elements of the intervention need to be considered separately in a future study. Finally, the effects of the nitrous oxide sedation per se are another factor that cannot be separated from the KomHIT communication intervention. A future study controlling this factor and comparing it to communication intervention would be of great value.

## 6. Conclusions

The results showed that the intervention had a positive effect in significantly lowering the level of preprocedural distress. The results showed that the pain intensity was lowered however not reaching statistical significance, and children reported low levels of pain intensity. This confirms other positive research results on the use of visual supports within paediatric care, a topic that has to be further studied.

## Figures and Tables

**Table 1:** Demographic data.

	Preinterventional (numbers)	Postinterventional (numbers)
Participants	50	50
Planned visits	39	32
Acute visits	11	18
Girls	24	24
Boys	26	26
Age (years)	9.64	9.12
Lumbar puncture	14	19
Joint injection	10	4
Pin removal	17	19
Others	9	8

**Table 2:** The differences of distress and pain intensity between preinterventional and postinterventional groups.

	Preinterventional mean (range, SD)	Postinterventional mean (range, SD)	*p* value
FAS (0.04–0.97) before	0.52 (0.04–0.97, 0.27)	0.41 (0.04–0.85, 0.28)	0.04^*∗*^
FAS during	0.32 (0.04–0.97, 0.28)	0.33 (0.04–0.85, 0.28)	0.85
FAS after	0.25 (0.04–0.85, 0.24)	0.19 (0.04–0.85, 0.20)	0.16
CAS (0–10) before	1.19 (0–8, 2.17)	0.62 (0–6, 1.39)	0.12
CAS during	1.67 (0–9, 2.52)	1.74 (0–8, 2.28)	0.88
CAS after	0.90 (0–9, 1.96)	0.42 (0–5, 1.08)	0.13

^*∗*^ *p* < 0.05.
